# Insights into Thermophilic Plant Biomass Hydrolysis from *Caldicellulosiruptor* Systems Biology

**DOI:** 10.3390/microorganisms8030385

**Published:** 2020-03-10

**Authors:** Sara E. Blumer-Schuette

**Affiliations:** Department of Biological Sciences, Oakland University, Rochester, MI 48309, USA; blumerschuette@oakland.edu

**Keywords:** *Caldicellulosiruptor*, extreme thermophile, genomics, thermostable enzymes, enzyme synergy, systems biology

## Abstract

Plant polysaccharides continue to serve as a promising feedstock for bioproduct fermentation. However, the recalcitrant nature of plant biomass requires certain key enzymes, including cellobiohydrolases, for efficient solubilization of polysaccharides. Thermostable carbohydrate-active enzymes are sought for their stability and tolerance to other process parameters. Plant biomass degrading microbes found in biotopes like geothermally heated water sources, compost piles, and thermophilic digesters are a common source of thermostable enzymes. While traditional thermophilic enzyme discovery first focused on microbe isolation followed by functional characterization, metagenomic sequences are negating the initial need for species isolation. Here, we summarize the current state of knowledge about the extremely thermophilic genus *Caldicellulosiruptor*, including genomic and metagenomic analyses in addition to recent breakthroughs in enzymology and genetic manipulation of the genus. Ten years after completing the first *Caldicellulosiruptor* genome sequence, the tools required for systems biology of this non-model environmental microorganism are in place.

## 1. Introduction

Over 50 years have passed since Thomas Brock’s discovery that Yellowstone National Park’s hot springs were teeming with microbial life [1]. In the half century after his seminal research, advances in DNA sequencing and molecular cloning techniques have hastened the discovery of enzymes from extreme to hyperthermophilic microorganisms. Heterotrophs that thrive at these elevated temperatures are of special interest for their robust and temperature-stable carbohydrate active enzymes (CAZymes) capable of deconstructing even the most recalcitrant parts of plant biomass [2,3]. Prior to the 1980s, moderate thermophiles, such as *Hungateiclostridium thermocellum* (formerly ‘*Clostridium thermocellum*’), were isolated and described as being capable of rapidly degrading cellulose [4], followed by the discovery of macromolecular enzyme complexes known as cellulosomes [5,6]. The question remained, however, if microorganisms inhabiting terrestrial hot springs at higher temperature ranges were capable of degrading all carbohydrates in plant biomass similar to *H. thermocellum*. Reports of cellulolytic microbial isolates enriched from terrestrial hot spring samples grown above 70 °C [7,8,9,10] raised the upper temperature limits for biological crystalline cellulose hydrolysis.

Bacterial lignocellulose deconstruction at elevated temperatures (above 70 °C) evolved to leverage specific carbohydrate active enzyme (CAZyme) families and carbohydrate binding modules (CBM) [3]. Enzymes capable of hydrolyzing glycosidic bonds within cellulose chains (endo-acting) have been characterized from hyperthermophilic microoganisms [11,12,13], however, cellobiohydrolase enzymes (exo-acting) have been identified in microorganisms growing optimally up to a temperature range of 70 to 80 °C, which appears to be the thermal limit for true cellulase activity. Possibilities for this observed phenomenon include the lack of crystalline cellulose in hyperthermophilic marine biotopes, or alternatively, that cellobiohydrolases evolved in the terrestrial extreme thermophiles and do not share a common ancestry with hyperthermophiles as a result. Evidence of horizontal gene transfer of primary cellulases between extreme and hyperthermophilic microbes has yet to be observed. Therefore, extremely thermophilic bacteria remain as the main source of the most thermostable primary cellulases on Earth.

## 2. Maturation of *Caldicellulosiruptor* Genomics

With the discovery of extremely thermophilic, plant biomass deconstructing genus *Caldicellulosiruptor*, the original focus was the function of modular, multi-functional enzymes that were cloned and sequenced (originally reviewed in [14]). However, the full diversity of the *Caldicellulosiruptor* enzymatic capacity was not fully appreciated until the first genome was published in 2008 [15]. Adding to the interest in this genus, an additional 40 genome sequencing projects involving the genus *Caldicellulosiruptor* were reported on the genomes online database [16], consisting of a total of 14 species. Since the release of the *C. saccharolyticus* genome in 2008, the average annual number of peer reviewed publications on the genus *Caldicellulosiruptor* has increased roughly 13-fold. The availability of the genome sequences catalyzed a sharp increase in knowledge on the enzymology and physiology of this genus.

### 2.1. Comparative Caldicellulosiruptor Genomics

The initial comparison between *Caldicellulosiruptor saccharolyticus* and *Caldicellulosiruptor bescii* (formerly ‘*Anaerocellum thermophilum*’) identified that the glucan degradation locus (GDL) could vary in complexity between strongly cellulolytic species [17]. Based on earlier shotgun cloning efforts to identify genes located in the glucan degradation locus (GDL), it was already observed that *C. bescii* encoded for a number of cellulases and xylanases [18], and that at least one of these enzymes was a multifunctional, modular cellulase [19] that shared similarities to CelA encoded by *C. saccharolyticus* [20]. Overall, both *C. saccharolyticus* and *C. bescii* possess a high number of carbohydrate binding modules and glycoside hydrolases in their respective genomes when compared to other thermophiles, and over 86% of the open reading frames (ORFs) in their respective genomes were orthologs [17].

As expected, the *Caldicellulosiruptor* core genome has been reduced from 1543 open reading frames (ORFs) shared among eight species [21] to 1367 ORFs shared among 14 species [22]. In comparison, the high level of genetic diversity among the genus *Caldicellulosiruptor* led to an increase of the pangenome from 3493 ORFs (13 species) [23] to 3791 ORFs (14 species) [22]. The initial *Caldicellulosiruptor* pangenome was constructed from the genomes of eight strongly to weakly cellulolytic members of the genus *Caldicellulosiruptor* [24,25], and was analyzed along with proteomics data to identify key genes unique to the strongly cellulolytic members of the genus *Caldicellulosiruptor*, that would assumedly be crucial to their strongly cellulolytic lifestyle [21]. 

#### 2.1.1. Evolutionary Adaptations to a Strongly Cellulolytic Lifestyle

While most species had been isolated and described as being cellulolytic (see Table 1) when tested for their ability to shred filter paper, some species did not degrade filter paper to any appreciable degree [21,25] and were excellent candidates for comparison to the strongly cellulolytic species. The presence of enzymes possessing a catalytic glycoside hydrolase (GH) family domain (including CelA) were determined to be a marker of cellulolytic capacity [21]. Ten of the 14 sequenced *Caldicellulosiruptor* genomes encode for enzymes containing at least one GH48 domain, with six of those species encoding for two (*C. obsidiansis* and *C. changbaiensis*) or three GH48 domains (*C. bescii*, *C. danielii*, *C. kronotskyensis*, *C. morganii*, and *C. naganoensis*) [21,22,23]. Regardless of the number of GH48 domains, most cellulolytic *Caldicellulosiruptor* species grow at similar rates on microcrystalline cellulose. However, their overall ability to solubilize biomass or crystalline cellulose varies, with *C. bescii* and *C. morganensis* outperforming other cellulolytic species [22,23,26].

This variability in enzymatic capacity may be related to the functional role each species plays during plant biomass degradation in situ. Accordingly, in a physiological comparison between two highly (*C. bescii*, *C. kronotskyensis*) and one moderately cellulolytic species (*C. saccharolyticus), C. saccharolyticus* was observed to more efficiently ferment the limited amount of soluble sugars released by its enzymes in comparison to the highly cellulolytic *Caldicellulosiruptor* species [26]. Furthermore, the total number of CAZymes does not correlate with cellulolytic ability, as the highly cellulolytic *C. kronotskyensis* and weakly cellulolytic *C. hydrothermalis* have the highest and second highest number of glycoside hydrolase (GH) domains [21]. Most of the observed diversity in GH domains is due to non-GDL genes, although new combinations of GH domains in GDL-encoded modular enzymes were noted for *C. morganii*, *C. danielii*, and *C. naganoensis* [23].

With the availability of genome sequences, custom oligonucleotide microarray chips were designed for *C. saccharolyticus* [15], *C. bescii* [17], and *C. kronotskyensis* [26]. Most studies focused on comparisons of global transcriptomes in response to various monosaccharide sugars [15,28], plant related polysaccharides [17,28,29], and plant biomass [26,29]. Despite their common ability to solubilize plant biomass, differences in the regulation of CAZymes, motility-related genes, and ABC transporters have been observed among the highly cellulolytic *C. bescii*, *C. kronotskyensis*, and moderately cellulolytic *C. saccharolyticus* [26] and in a proteomic comparison between *C. bescii* and *C. obsidiansis* [30]. Together, these studies imply that within *Caldicellulosiruptor* communities, even species producing powerful cellulases may specialize within their community. *C. bescii* and *C. kronotskyensis*, for example, were both isolated from the Kamchatka, Russia region [10,31], and *C. saccharolyticus* was isolated from the Taupo region of New Zealand, along with other phenotypically different members of the same genus [32].

#### 2.1.2. Diverse Mechanisms Used to Maintain Cell-Substrate Proximity

Aside from using comparative genomics to determine the cellulolytic capacity of the genus *Caldicellulosiruptor*, each species was found to differ in the number of genes encoding for proteins and enzymes involved in the attachment to lignocellulosic substrates. Proteins with S-layer homology (SLH) domains are one such class and are required for the interface between cellulosomes and cells in the Clostridiales [33]. However, in the non-cellulosomal genus *Caldicellulosiruptor*, each species sequenced possesses multiple S-layer located enzymes and proteins (between 10 and 19) [34]. Interestingly, the number of genes encoding for S-layer located proteins does not correlate with cellulolytic capacity [21], including the distribution of S-layer enzymes [34]. One S-layer located enzyme is a part of the *Caldicellulosiruptor* core-genome (GH5-CBM28-SLH-SLH-SLH) [21,34] and was proposed to function as an enzyme and, additionally, to promote adherence to cellulose [35]. Latter studies identified that other S-layer proteins unique to various *Caldicellulosiruptor* species were also involved in the attachment to cellulose and pectin [35] or xylan [34]. 

Lastly, high-temperature cellulose adhesins (tāpirins) were identified through comparative genomics and proteomics efforts [21,36]. Genes encoding for uncharacterized hypothetical proteins, later identified as tāpirins, are located directly upstream of the GDL, and were initially demonstrated to have micromolar affinity for microcrystalline cellulose along with an unusual β-helix shape [36]. Two additional tāpirin structures were recently solved from divergent tāpirins produced by weakly cellulolytic species (*C. hydrothermalis* and *C. kristjanssonii*) that share a similar shape to the highly cellulolytic tāpirin proteins [37]. Furthermore, biophysical analysis of tāpirins from weakly cellulolytic species indicates that these tāpirins bind to more sites on cellulose, likely aided by a longer predicted binding pocket in their structure [36,37], and grant a competitive advantage to adhere to plant biomass in their environment. Not all strongly cellulolytic members of the genus *Caldicellulosiruptor* use classical tāpirins to adhere to cellulose, as atypical tāpirin proteins similar to those encoded for by *C. owensensis* and *C. acetigenus* were identified in the genome of *C. changbaiensis* [22]. Additional biophysical analysis of these atypical tāpirins is required, as they may represent a unique mechanism of cellulose adherence used by this genus.

### 2.2. Caldicellulosiruptor Community Analyses

**Figure 1:**
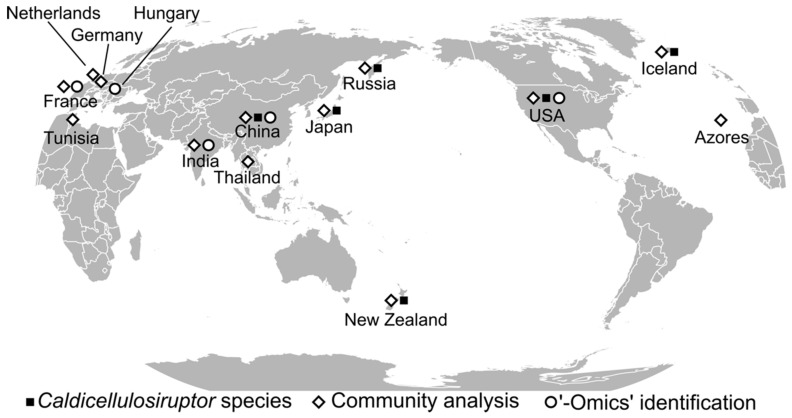
Global sites where members of the genus *Caldicellulosiruptor* have been isolated or detected. Locations are approximate to country or territory from peer-reviewed literature (see Table 2). Open diamonds, 16S rRNA gene sequencing; open circles, meta-omics identification; closed squares, *Caldicellulosiruptor* spp. genomic sequencing.

The goal of enriching extremely thermophilic cellulolytic microorganisms was to ultimately identify thermostable enzymes from those organisms. Prior to the isolation of individual species, communities of cellulolytic microbes from geothermally heated springs were enriched on cellulose [7,9,38,39] for initial biochemical and phylogenetic analysis. At that time, New Zealand was the site of the most extensive phylogenetic characterization of *Caldicellulosiruptor* strains [32]. However, due to the amplicon length constraints of Sanger sequencing, initial CAZymes identified from the genus *Caldicellulosiruptor* were identified from isolated species, such as *C. bescii* [19] and *C. saccharolyticus* [20,40,41,42], rather than from cellulolytic communities. Development of primer pairs specific for conserved regions of 16S rRNA genes (reviewed in [43]) allowed for culture-independent insights into the global presence of the genus *Caldicellulosiruptor* (see Figure 1). Furthermore, the ability of the genus *Caldicellulosiruptor* to hydrolyze plant biomass or other recalcitrant polysaccharides and produce acetate and hydrogen at elevated temperatures [44,45,46,47] has led to the identification of environmental strains in fermenters and digesters inoculated with environmental samples. 

The availability of genome sequences has bolstered *Caldicellulosiruptor* species identification from hot springs microbial community analyses from the Azores [48], Iceland [49], Russia [50], Tunisia [51], and the United States [23,52] (see Table 2). While *Caldicellulosiruptor* species were isolated from most sites sampled, the only report of *Caldicellulosiruptor* from the African continent was a single 16S rRNA gene clone identified from a hot spring in Tunisia, which was predominantly colonized by Firmicutes [51]. Based on culture-independent 16S rRNA gene amplification and metagenome sequencing, the genus *Caldicellulosiruptor*, along with genera *Fervidobacterium*, *Dictyoglomus*, and *Sulfurihydrogenibium* were most abundant in a slightly alkaline, highly reduced solfatara effluent stream in the Azores [48]. The genera *Caldicellulosiruptor*, *Fervidobacterium*, and *Dictyoglomus* were also detected by 16S rRNA gene clones isolated from separate enrichment cultures sampled from springs in the Uzon Caldera, Kamchatka, Russia [50]. Obsidian Pool in Yellowstone National Park in the United States has been extensively sampled, including two high-temperature enrichment studies focused on plant-biomass degrading heterotrophs [23,52]. In both studies, the genus *Caldicellulosiruptor* predominated enrichment cultures between 70 and 75 °C [23,52]. Community analysis also identified *Caldicellulosiruptor* species in extremely thermophilic hydrogen- or methane-producing consortia that were sourced from the Ta Na Ma Rao hot spring in Thailand [53]. One study also yielded a novel β-galactosidase enzyme cloned from an uncultured *Caldicellulosiruptor* species present in a hot spring from Yongtai, China, that was identified through 16S rDNA phylogeny [54]. Overall, cellulose enrichment cultures from multiple continents grown above 70 °C were composed predominantly of members from the genus *Caldicellulosiruptor*, supporting their primary role in cellulose hydrolysis at elevated temperatures. Interestingly, geothermally heated springs are not the only potential sources of isolated *Caldicellulosiruptor* species, for example, *C. owensensis* was isolated from solar heated mud flats in Owens Lake, CA [55], and *C*. sp. F32 was isolated from a compost enrichment in China [56]. *Caldicellulosiruptor* spp. most similar to *C. kristjanssonii* based on 16S rDNA analysis were identified from manure-supplemented distillery waste streams [57], although it is unclear if the *Caldicellulosiruptor* cells came from the distillery wastewater or the digested cow manure. Additional reports of *Caldicellulosiruptor* species identified from animal microbiota included community analyses of a rumen microbial community [58], and poultry caecum community [59] opening up the exciting prospect of mesophilic relatives of the *Caldicellulosiruptor* (“*Mesocellulosiruptor*”), similar to the discovery of mesophilic members of the phylum *Thermotogae* (reviewed in [60]). *Caldicellulosiruptor* species were also detected in the latter stages of a human waste-amended thermophilic aerobic digestor in Japan [61], and by community analysis from anaerobic digesters present in France [62], India [63], China [64], and Hungary [65]. The anaerobic digester in Hungary was run at mesophilic temperatures, and was initially inoculated with pig manure, further supporting the existence of mesophilic *Caldicellulosiruptor* as part of animal gut microbiomes. Finally, other studies on fermenters inoculated with soil from Germany [66], and a hydrogen-generating bioreactor in the Netherlands with unknown inoculum [67] also identified different *Caldicellulosiruptor* species than those used in the inoculum, *C. saccharolyticus*. Further lines of evidence supporting the existence of “*Mesocellulosiruptor*” include a recent survey of soil microorganisms in Spain that identified *Caldicellulosiruptor* species as minor members of the soil community [68]. Of all the reports of *Caldicellulosiruptor* species, the most unusual may be of the identification of a *C. lactoaceticus* 16S rRNA gene clone from drilling fluid sampled roughly 2.5 km below the Earth’s surface [69]. Due to the multiple examples of *Caldicellulosiruptor* 16S rRNA genes being identified in bioreactors, animal manure, and soil, biotopes, aside from geothermally heated springs, appear to harbor *Caldicellulosiruptor* species, or relatives of the genus (“*Mesocellulosiruptor*”) that may also possess robust enzymes of interest.

### 2.3. Caldicellulosiruptor Meta(proteo-)genomics

With rapid advancements in next generation sequencing and proteomics, an assembly of partial genomes or identification of active metabolic pathways from environmental *Caldicellulosiruptor* isolates present in microbial communities is possible. Interestingly, the initial studies using next generation ‘-omics’ techniques that identified *Caldicellulosiruptor* species as predominant members of microbial communities started from anaerobic digestion sludge [62,65]. For example, *Caldicellulosiruptor* spp. were identified through metaproteomics as one of the predominant bacteria involved in polysaccharide hydrolysis in a cellulolytic microcosm that was initially inoculated with anaerobic digester sludge [62]. Analysis of whole cell protein fractions identified peptides corresponding to the predominant *Caldicellulosiruptor* cellulase, CelA, along with CAZymes, more commonly associated as being intracellular [62]. It is not surprising that few extracellular *Caldicellulosiruptor* enzymes were identified in this study since neither the supernatant nor substrate were sampled for peptide identification. Other proteomic efforts on the genus *Caldicellulosiruptor* have highlighted the differences in the peptide profiles of whole cell versus supernatant [30,72] or between whole cell, supernatant, and substrate-bound samples [21].

In addition to the identification of hydrolytic enzymes from anaerobic digestor metaproteomics, metagenomes were sequenced from enriched cellulolytic communities harvested from Obsidian Pool, Yellowstone National Park in the United States [23]. While a sole *Caldicellulosiruptor* species was previously isolated from Obsidian Pool (*C. obsidiansis*), other global sites, New Zealand, for example, host a diverse community of *Caldicellulosiruptor* species [73]. Cellulolytic communities, enriched in situ or from water samples, were sequenced to assess the diversity of *Caldicellulosiruptor* species in other hot spring environments. Initial community analysis of these cellulose-enriched communities determined that members of the *Caldicellulosiruptor* genus ranged from 89.9% to 38.7% of the total community population, although the relative abundance of *Caldicellulosiruptor* species only marginally correlated with solubilization of lignocellulose [23], emphasizing the role of other non-cellulolytic members of these microbial communities. Incomplete *Caldicellulosiruptor* genomes assembled from metagenomes shared a high degree of homology and synteny with the *C. obsidiansis* genome. Regardless, key differences were noted in their GDL composition, including the presence of an additional modular cellulase (GH10-CBM3-CBM3-GH48) in two of the enrichments that *C. obsidiansis* lacks [23]. These communities were enriched on microcrystalline cellulose prior to metagenomic sequencing, which may have resulted in an overrepresentation of the strongly cellulolytic *Caldicellulosiruptor* strains, considering that half of the communities enriched encoded for the three-key synergistic cellulases, CelA (GH9-CBM3-CBM3-CBM3-GH48), CelC (GH10-CBM3-CBM3-GH48), and CelE (GH9-CBM3-CBM3-CBM3-GH5) [74]. 

## 3. Modular Caldicellulosiruptor CAZymes

Every strongly cellulolytic member of the genus *Caldicellulosiruptor* that has been genome sequenced encode for a locus of modular, multifunctional enzymes. This genomic region, named the glucan degradation locus (GDL), contains one or more enzymes that possess the marker for the highly cellulolytic phenotype, GH 48 [21,25]. Historically, cellulases from the genus *Caldicellulosiruptor* were under considerable focus, and the first report of large (>180 kDa) glycosylated cellulases was noted from *C. naganoensis* (formerly ‘*Thermoanaerobacter cellulolyticus*’) [75]. The modular architecture of enzymes encoded by the GDL was first reported after sequencing a genome library clone that encoded for *celB* (GH10-CBM3-GH5) from *C. saccharolyticus* [76], which was then rapidly followed by the cloning and characterization of other modular enzymes from *C. saccharolyticus* [41,42]. Initially, the identification of modular enzymes encoded by the GDL or xylan degradation locus (XDL) was facilitated by sequencing lambda phage genomic libraries constructed from *Caldicellulosiruptor* sp. Tok7B.1 [14,77] or *C. saccharolyticus* [20,78]. 

Now, with the benefit of comparative genomics including 14 *Caldicellulosiruptor* genomes, and one metagenome assembled genome [21,22,23], the diversity of GH architecture in the GDL is well characterized, and the minimal complement of cellulases required for cellulose or lignocellulose hydrolysis has been identified [74,79]. From newly available genome sequences, modular enzymes present in the GDL were identified with new combinations of catalytic domains or additional CBM3 modules in established modular enzymes, such as the case with *C. morganii* [23]. The enzymatic significance of these changes still needs to be characterized, although enzymes with the same modular structure, CelA, for example, have different activities, with CelA from *C. bescii* outperforming CelA from *C. danielii* [23]. Considering that only CelA from *C. saccharolyticus* [20], *C. bescii* [19,80,81], and now *C. danielii* have been characterized, it remains to be seen if there is a superior candidate to *Cb*CelA. As individual enzymes from various *Caldicellulosiruptor* species have been cloned, each research group has used differing gene naming conventions. In this review, the naming convention for the GDL in *C. bescii* will follow unless noted.

### 3.1. CelA

While heterologous expression in *Escherichia coli* is commonplace for the initial characterization of recombinant enzymes, the size of the modular cellulases may be a limiting factor for the adoption of these cellulases in other systems. The largest *Caldicellulosiruptor* modular enzyme, CelA (GH9-CBM3-CBM3-CBM3-GH48), was initially identified from screening a *C. saccharolyticus* genome library for activity on amorphous cellulose, and was subsequently sequenced [20]. However, hydrolysis of crystalline cellulose was not observed until natively purified CelA was characterized from *C. bescii* [19]. Furthermore, observation of CelA-treated microcrystalline cellulose particles highlighted this enzyme’s unique mechanism of tunneling through cellulose fibers during hydrolysis, in addition to the more commonly observed chewing back mechanism that other commercial cellulases display [81]. Both catalytic domains of CelA likely coordinate efficient hydrolysis through intramolecular synergy (GH9, endoglucanase, and GH48, cellobiohydrolase) as demonstrated by the intermolecular synergy of single GH truncations during hydrolysis of microcrystalline cellulose [80]. While CelA is capable of outperforming commercial fungal cellulase mixtures [81] and can hydrolyze highly crystalline cellulose, it will adsorb to lignin, rendering the enzyme ineffective [82]. Interestingly, *C. bescii* was observed to solubilize all components of plant biomass, lignin included, during growth on switchgrass, which could alleviate CelA inhibition by lignin adsorption [83]. However, this phenomenon is likely dependent on the plant species, as *C. bescii* was unable to solubilize lignin present in poplar [84].

Contribution of CelA to cellulose hydrolysis was also demonstrated by a *C. bescii celA* knockout mutant, which was reduced in its ability to grow on cellulose and overall cellulase activity when induced by cellobiose [85]. In contrast, deletion of *celA* in a genetically stable *C. bescii* parental strain [86] only resulted in a reduction in cellulolytic ability by 45% [74]. One explanation for this discrepancy is misassembled regions in the original *C. bescii* genome (NCBI accession NC_012034.1) [86] used to design the original CelA knockout vector in Young et al. [85]. These assembly errors in the GDL are not entirely unexpected, as there are multiple repeating nucleotide sequences present in the GDL particularly those encoding for carbohydrate binding modules and GH family 48 and 5 domains [74]. Primers designed for the initial *celA* knockout vector will amplify a 1 kb downstream sequence that is present in multiple regions and could result in the deletion of an additional two GDL genes (*celB*, and *celC*). Expansion and diversity of the *Caldicellulosiruptor* GDL has been proposed to occur through gene recombination after duplication [14,77] and would explain the presence of these repeated regions in the GDL. This deletion strain is still useful for the in vitro analysis of CelA enzyme engineering, however, and directed mutations of native *C. bescii celA*, by insertion of N-terminal aspartate repeats, improved glucose release by the extracellular protein fraction [87] and improved growth on cellulose as monitored by plate counting [85].

Recent characterization of full length and truncated CelA has used natively purified enzyme [81], native overexpression [74,79,88,89], and heterologous expression in *E. coli* or *Bacillus megaterium* [80], where the results from homologously versus heterologously produced CelA may not be comparable, due to differences in post-translational modifications, such as glycosylation. Linker regions present in modular enzymes are rich in proline–threonine repeats that indicate that CelA is likely glycosylated, and one report on the native purification of CelA indicated that the purified protein stained positively for glycosylation [19]. Glycosylation of CelA with galactose disaccharides was confirmed by overexpression in the *C. bescii ΔcelA* strain [89,90], only extracellular CelA was observed as being glycosylated [89] and the glycotransferase (GT) located in the GDL is required for CelA glycosylation [79,91]. Initially, deletion of the GT responsible for glycosylation of CelA resulted in proteolytic clipping of extracellular enzymes and lower cell densities observed by plate counts from planktonic cells growing on microcrystalline cellulose [91]. In contrast, deletion of the same GT from a different parental *C. bescii* strain did not result in a dramatic effect, and the GT deletion strain was still capable of solubilizing microcrystalline cellulose [79]. Glycosylation of CelA does have a protective effect against proteases, and full length glycosylated CelA hydrolyzed 22% more microcrystalline cellulose in vitro versus non-glycosylated CelA [90]. In contrast, Conway et al. monitored cellulose solubilization in vivo, and other factors aside from glycosylation may impact enzyme efficiency [79]. Lastly, while fewer *C. bescii* GT knock out colonies were observed by Russell et al., [91], no comparison in growth on a solid medium was made between planktonic cells grown on cellobiose versus cellulose. It is possible that in addition to reduced stability of extracellular hydrolytic enzymes, the glycosylation deficient *C. bescii* strains are also deficient in their ability to form colonies on solid medium, resulting in a false negative result for growth on microcrystalline cellulose. 

### 3.2. Modular Cellulase Synergy

In addition to the synergy between catalytic domains in the modular cellulases, it would be expected that the enzymes themselves work in concert to hydrolyze plant biomass. Synergism between heterologously produced (*E. coli*) truncations of CelB (*-CBM3-CBM3-GH44), with CelE (GH9-CBM3-CBM3-CBM3-*), CelD (*-CBM3-CBM3-CBM3-GH5), or the S-layer located cellulase (GH5-CBM28-*) was detected [92]. Interestingly, the combination of truncated CelE with CelD was only additive in activity, while homologously produced full-length CelE and CelD synergistically hydrolyzed microcrystalline cellulose [79]. When full-length modular cellulases from the GDL were tested for synergistic activity, most combinations were found to be positive, highlighting the need for homologously produced full length enzymes. The combination of CelA, CelC (GH10-CBM3-CBM3-GH48), and CelE was most optimal (degree of synergy, 3.8) on a glucan conversion basis when compared to mixtures of CelA plus three or more additional enzymes from the GDL [79]. In contrast, when monitoring the activity of the GDL enzymes in vivo, the deletion of CelA, CelB, and CelC was sufficient to reduce cellulose solubilization by over 90% [74]. Regardless of the activity of cell-free cellulase enzyme mixtures, the highest levels of cellulose solubilization were observed when *C. bescii* cells are present [79], supporting past findings in other systems that cell-substrate proximity also plays a role in efficient cellulose hydrolysis [93]. Four additional combinations of catalytic domains in the GDL were identified from the genome sequencing of *C. danielii*, *C. morganii*, and *C. naganoensis*, including two enzymes that include three catalytic domains (GH12-Gh5-CBM3 × 3-GH48 and GH10-CBM3-GH12-GH48) [23]. Homologous production of full length, glycosylated versions of these enzymes noted that these enzymes, like others encoded by the *C. bescii* GDL, hydrolyze microcrystalline cellulose synergistically [94] and are candidates for engineering a superior *C. bescii* biocatalyst.

### 3.3. Xylanases 

In addition to the GDL, six isolated species, *C. acetigenus*, *C. bescii*, *C. changbaiensis*, *C. kronotskyensis*, *C. owensensis,* and *C. saccharolyticus* encode for a second locus of modular enzymes capable of hemicellulose hydrolysis [21]. While not as extensively studied, synergy exists between the main xylanases and side-chain cleaving enzymes from *C. bescii* [95]. Other, less cellulolytic *Caldicellulosiruptor* species may be superior sources of xylanases that will work synergistically with cellulases, since they have evolved to specialize on the other carbohydrate components of plant biomass. One example are the extracellular enzymes from weakly cellulolytic *C. owensensis*, which significantly increased the amount of sugar released from non-pretreated, high-hemicellulose plant biomass when used sequentially with commercial cellulase mixtures [96]. The exact polysaccharide composition of xylan can change between plant species, and more than one type of xylanase may be required to efficiently hydrolyze this heterogenous polysaccharide. An S-layer localized GH10 xylanase cloned from *C. lactoaceticus* was noted for five-fold higher activity on oat spelt xylan in comparison to birchwood or sugar case xylan [97]. It is foreseeable that future engineering efforts of *C. bescii* may be the inclusion of additional xylanases and other hemicellulose hydrolyzing enzymes from the more xylanolytic members of the genus, or other species, for example, *Acidothermus cellulolyticus*. Overexpression of two different xylanases from *A. celluloyticus* resulted in a significant improvement of *C. bescii* growth on birchwood xylan and sugar release from oat spelt or birchwood xylan [98], which were far larger gains than observed from a chromosomal knock-in of a *C. kronotskyensis* S-layer located xylanase in *C. bescii* [34]. 

### 3.4. Heterologous Expression of Caldicellulosiruptor Enzymes in Other Systems

Other thermophilic genera also serve as promising consolidated bioprocessing platforms, like *H. thermocellum* which is notable for its cellulosomes (reviewed in [99]) and cellulolytic efficiency [100], but is subject to feedback inhibition by cellobiose [101]. Prior studies have relieved this feedback inhibition in vitro by exogenous mixing of purified cellulosomes and chimeric native β-glucosidases with cohesion domains [101], or with other thermophilic β-glucosidases [102]. Since the genus *Caldicellulosiruptor* is extremely thermophilic, their enzymes, in theory, should prove to be stable when heterologously expressed in a system like *H. thermocellum*. Recently, a β-glucosidase from *Caldicellulosiruptor* sp. F32 was produced heterologously in *H. thermocellum* as a fusion to the cellulosomal cellobiohydrolase, CelS [103] where it increased cellulose hydrolysis best when expressed as a chromosomal knock-in rather than from a replicating plasmid. Interestingly, in a follow-up study, when the same β-glucosidase was fused to a cohesin type II domain and incorporated into the cellulosome, it continued to act synergistically in the hydrolysis of microcrystalline cellulose, and hydrolyzed wheat straw at better rates than non-supplemented cultures [104]. However, when crystallinity of the remaining cellulose was used as a measure of cellulase efficiency, a negative effect of cellulosomal β-glucosidase on hydrolysis was noted [104]. This is in contrast to a synthetic cellulosome constructed from recombinant enzymes and scaffoldin which incorporated a *C. saccharolyticus* β-glucosidase [105]. In this case, the β-glucosidase was fused to a dockerin domain similar to native cellulosomal enzymes, and a synergistic effect during corn stover hydrolysis was observed between the β-glucosidase from *C. saccharolyticus* and endo-cellulase and xylanases from thermophilic *Geobacillus* strains [105]. More recently, a designer cellulosome was constructed from all *C. bescii* catalytic domains, GH9 and GH48 from CelA, and GH5 from the core S-layer endoglucanase that outperformed (>1.5-fold sugar release) the native *H. thermocellum* cellulosome during extended cellulose hydrolysis times at 75 °C [106], demonstrating proof of concept for hyperthermophilic cellulosomes.

In addition to heterologous expression in other Firmicutes, *Caldicellulosiruptor* modular cellulases, CelA (Csac_1076) and CelB (GH10-CBM3-GH5, Csac_1078), from *C. saccharolyticus* were introduced and expressed in *Thermotoga* sp. RQ2 [107], which resulted in a modest increase in detectable endoglucanase activity. A truncated form of ‘*celB*’ from *C. saccharolyticus* (*-CBM3-GH5) was included in an ionic liquid (IL)-tolerant enzyme cocktail (JTherm) for concurrent saccharification and IL treatment, which released over three times more sugars from IL-pretreated biomass at elevated temperatures [108]. The same CelB truncation was heterologously expressed in the fungus, *Aspergillus niger*, and was again able to retain a high level of activity in comparison to other bacterial EGs expressed [109]. This is not surprising as two unidentified EG clones from a *C. naganoensis* genomic library [110,111] were also heterologously expressed in the fungus *Saccharomyces cerevisiae* [112,113] and released more sugars than the comparable clones expressed in *E. coli*.

## 4. Caldicellulosiruptor Genetic Engineering

In parallel with the intense enzymatic focus on the genus *Caldicellulosiruptor*, the development of genetic tools afforded the opportunity to develop *C. bescii* as a potential platform organism for consolidated bioprocessing (CBP) where the same organism would enzymatically attack and ferment plant biomass to usable products (CBP reviewed in [114,115]). Development of the *Caldicellulosiruptor* genetic system relied on the identification of restriction–modification systems [116] that were impeding transformation by improperly methylated DNA isolated from *E. coli* [116,117]. Genetic systems for *C. bescii* now include a *pyrF*-based counter selection method [118,119] or kanamycin selection based on a highly thermostable resistance gene [120] together with a genetically tractable strain of *C. bescii* that experiences less mobile element genome rearrangements [86]. Reverse genetics using the *pyrF*-based counter selection method initially demonstrated the impact of CelA [85] and a downstream pectinase cluster [121] on the ability of *C. bescii* to grow on plant polysaccharides. In addition to the development of reverse genetics systems, an *E. coli*–*C. bescii* shuttle vector was constructed using the origin of replication from pBAS2 [122], one of the two native *C. bescii* plasmids [123]. Aside from being used for protein overexpression in *C. bescii*, this shuttle vector was also successfully maintained by *H. thermocellum* [124], expanding genetic tools for other thermophilic Firmicutes. 

### 4.1. Metabolic Engineering

There is a large body of work investigating anaerobic hydrogenesis by the genus *Caldicellulosiruptor* (reviewed in [125]), including intriguing recent findings of diauxic-like hydrogen productivity during co-fermentation of C_5_ and C_6_ sugars [126]. Conversion of plant biomass hydrolysates to hydrogen by *C. saccharolyticus* were previously demonstrated [47], and recent sequential co-cultures of *Caldicellulosiruptor* species coupled with mesophilic chemoorganotrophs [127] or chemolithotrophs [128] converting the *Caldicellulosiruptor* fermentation products to value added chemicals like polyhydroxybutyrate. 

Hydrogenesis also represents an attractive pathway to engineer in *C. bescii* for either increased productivity or redirection of reducing equivalents to other valuable metabolic pathways. Accordingly, the initial genetic manipulations of *C. bescii* were conducted with the goal of developing *C. bescii* as a suitable platform organism for biofuel production. Deletion of the lactate dehydrogenase gene (*ldh*) redirected reducing equivalents from the reduction of pyruvate to lactate to hydrogenases, which increased hydrogen gas production [119]. Furthermore, by introducing a replicating vector heterologously expressing an alcohol dehydrogenase gene (*adhE*) from *H. thermocellum*, *C. bescii* produced ethanol when grown on switchgrass with no exogenously added CAZymes [121]. This was a significant step, demonstrating that *C. bescii* was capable of second generation consolidated bioprocessing, where no physiochemical pretreatment of the plant biomass was required for fermentation. Further improvements in ethanol titers during the growth on cellulose or xylan were made by introducing a reduced ferredoxin NAD oxidoreductase complex (Rnf) from *Thermoanaerobacter* sp. X514 [129] into *C. bescii*, which increased the NADH/NAD^+^ ratio [130]. 

Since the optimal growth temperature of *C. bescii* (T_opt_ 78–80 °C) [131] is higher than that of *H. thermocellum* (T_opt_ 60 °C) [4], a bi-functional ADH was engineered into *C. bescii* from *Thermoanaerobacter pseudethanolicus* (T_opt_ 65 °C), which would likely perform better at the elevated growth temperatures used for *C. bescii* [132]. *T. pseudethanolicus* encodes for two bi-functional alcohol dehydrogenases, and between the two enzymes, *C. bescii* produced a higher titer of ethanol when expressing the NADH-dependent *T. pseudethanolicus* ADH during growth on microcrystalline cellulose or plant biomass [133]. However, the titers were almost 10-fold lower than those observed using the *H. thermocellum* ADH [121]. Another *T. pseudethanolicus* ADH, annotated as a butanol dehydrogenase, increased the tolerance of *C. bescii* to furfurals, a byproduct of thermal and acid-pretreatment of plant biomass, by reducing furfurals to their cognate alcohols which are less toxic [134].

### 4.2. Heterologous Expression of CAZymes

Aside from metabolic engineering, there also has been a great deal of interest in supplementing the CAZyme repertoire of *C. bescii* with additional, synergistic enzymes. Initially, modular *Caldicellulosiruptor* β-glucosidases were introduced in *H. thermocellum* to relieve cellobiose inhibition of cellulases [103,104,105], and in turn, two different enzymes, a β-glucosidase from *A. cellulolyticus* [135] and cellobiose phosphorylase [136], were produced in *C. bescii*, which increased the efficiency of cellulose hydrolysis. An important first step in increasing cellulolytic activity in *C. bescii* was demonstrated by chromosomal expression of a β-glucanase and/or endoglucanse from *A. cellulolyticus*, which resulted in synergistic increases in cellulose hydrolysis, and over a 17-fold improvement of *C. bescii* growth on cellulose [137]. Heterologous expression of a cellobiose phosphorylase from *Thermotoga maritima* to relieve cellobiose inhibition improved the performance of the *C. bescii* endoglucanase knock-in strain [91], and the best gains in cellulolytic capacity were observed when all three enzymes (β-glucanase, endoglucanse, and cellobiose phosphorylase) were co-expressed in *C. bescii* [138]. Clearly, even though the GDL of *C. bescii* had evolved to maximize synergy between the endogenous *C. bescii* enzymes, there are opportunities to engineer superior cellulolytic strains. Improvements in xylan solubilization were also noted for *C. bescii* xylanase knock-in strains. As described above, expression of multiple GH10 enzymes from *A. cellulolyticus* [98] or an S-layer located xylanase from *C. kronotskyensis* (Calkro_0402) in *C. bescii* increased xylan hydrolysis in both cases and increased xylan attachment by the *C. bescii* (Calkro_0402) knock-in strain [34].

## 5. Future Perspectives

**Figure 2:**
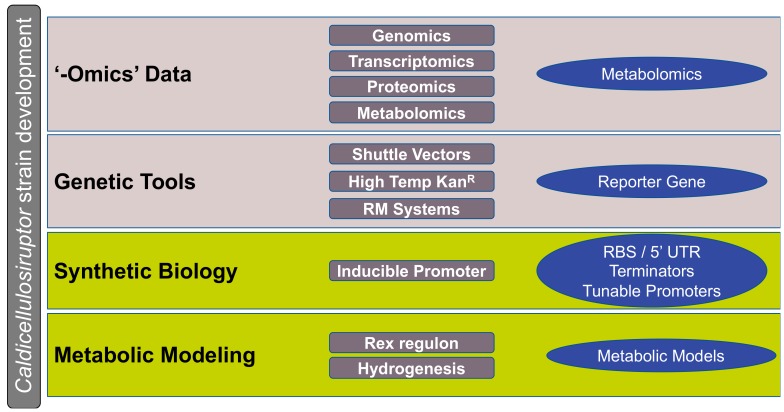
Progress on the generation of data and tools required for *Caldicellulosiruptor* strain development. Maturing research areas are shaded in light gray, nascent research areas are shaded in light green. Tools and data discussed in this review are listed in dark gray rectangles, tools that require further development for strain development are highlighted in the blue ovals. Image modified from strategies illustrated by Yan and Fong [135].

In the twelve years since the release of the first *Caldicellulosiruptor* genome, immense strides towards *Caldicellulosiruptor* strain development for consolidated bioprocessing have been made. While *Caldicellulosiruptor* enzymes are robust and have demonstrated promise when heterologously expressed in other systems [104,105,107,109,139,140,141,142], engineering *C. bescii* to produce value added chemicals from plant biomass is now more promising than ever. Critical developments, like the design of *C. bescii* genetic tools, now allow for reverse genetics and heterologous production of synergistic enzymes. Coupled with the previously established genomics, transcriptomics, and proteomics data available for the genus, the stage is set for further advancements towards targeted strain development. Initial reports of an inducible promoter [143] and identification of a redox-responsive regulon [144] in *C. bescii* are supporting the nascent field of *Caldicellulosiruptor* synthetic biology. Moving forward, the use of established ‘-omics’ tools to identify additional regulated promoters, optimal ribosomal binding sites, and other 5′ untranslated sequences will be critical for continued advancements in genetic manipulation of the genus *Caldicellulosiruptor* (see Figure 2).

## 6. Conclusions

Aside from lignocellulose deconstruction, the genus *Caldicellulosiruptor* is found to be increasingly versatile for other difficult biotransformations. Enzymes identified from the genus have been demonstrated to be useful for food and pharmaceutical production, in addition to textile and paper processing. Additionally, their fermentative physiology is now being investigated for the formation of metal nanoparticles [145,146] and heavy metal reduction [147]. More so now than ever with the benefit of genome sequences and the availability of functional genomics and genetics tools, this non-model environmental genus has the potential to move from the bench to a larger scale as a biotechnological platform microorganism.

## Figures and Tables

**Table 1:** Sequenced members of the genus *Caldicellulosiruptor.*

Species Name	Genome Size (Mb)	Total ORFs	GC%	Year Sequenced	Cellulolytic Capacity	Genome Reference
*C. acetigenus*	2.59	2498	36.1	2013	Weak	[23] ^a^
*C. bescii*	2.96	2905	35.2	2018 ^a^	Strong	[17]
*C. changbaiensis*	2.91	2532	35.1	2019	Strong	[22]
*C. danielii*	2.83	2731	35.8	2014	Strong	[23]
*C.* sp. F32	2.38	2487	35.2	2013	Strong	[27]
*C. hydrothermalis*	2.77	2685	36.1	2011	Weak	[21]
*C. kristjanssonii*	2.8	2707	36.1	2011	Weak	[21]
*C. kronotskyensis*	2.84	2642	35.1	2011	Strong	[21]
*C. lactoaceticus*	2.67	2549	36.1	2011	Moderate	[21]
*C. morganii*	2.49	2407	36.5	2014	Strong	[23]
*C. naganoensis*	2.51	2436	35.4	2014	Strong	[23]
*C. obsidiansis*	2.53	2389	35.2	2011	Strong	[24]
*C. owensensis*	2.43	2322	35.4	2011	Weak	[21]
*C. saccharolyticus*	2.97	2834	35.3	2007	Moderate	[15]

^a^ The publicly available genome for *C. acetigenus* was analyzed as a part of this study.

**Table 2:** Published *Caldicellulosiruptor* metagenomics or community analysis.

Country	Analysis	Origin Temperature	Source	Ref.
Azores	community analysis	Thermophilic	Geothermally heated springs	[48]
China	community analysis	Thermophilic	Anaerobic digester sludge	[64]
China	community analysis	Thermophilic	Drilling fluid	[69]
China	community analysis	Thermophilic	Geothermally heated spring	[54]
China	community analysis	Mesophilic	Unknown	[57]
France	metaproteomics, community analysis	Thermophilic	Anaerobic digester sludge, municipal solid waste	[62]
Germany	community analysis	Mesophilic	soil or compost	[66]
Hungary	metagenomics	Mesophilic	Pig manure	[65]
Iceland	cellulose or xylan enrichments	Thermophilic	Geothermally heated springs	[49]
India	metagenomic sequencing	Unknown	Anaerobic digester sludge	[63]
India	metagenomic and metatranscriptomic sequencing	Mesophilic	Gir cattle rumen	[58]
India	community analysis	Mesophilic	Poultry caecum	[59]
Japan	community analysis	Thermophilic	Thermophilic aerobic digester, human waste	[61]
Japan	community analysis	Thermophilic	Geothermally heated spring	[70]
Japan	community analysis	Mesophilic	Anaerobic sludge (upflow anaerobic sludge blanket)	[71]
Netherlands	community analysis	Thermophilic	Unknown	[67]
Thailand	community analysis	Thermophilic	Geothermally heated springs	[53]
Tunisia	community analysis	Thermophilic	Geothermally heated springs	[51]
United States	community analysis	Thermophilic	Geothermally heated spring	[52]
United States	metagenomic sequencing, community analysis	Thermophilic	Geothermally heated spring	[23]
